# Renormalization group approach to the Fröhlich polaron model: application to impurity-BEC problem

**DOI:** 10.1038/srep12124

**Published:** 2015-07-17

**Authors:** F. Grusdt, Y. E. Shchadilova, A. N. Rubtsov, E. Demler

**Affiliations:** 1 Department of Physics and Research Center OPTIMAS, University of Kaiserslautern, Germany; 2 Graduate School Materials Science in Mainz, Gottlieb-Daimler-Strasse 47, 67663 Kaiserslautern, Germany; 3 Department of Physics, Harvard University, Cambridge, Massachusetts 02138, USA; 4 Russian Quantum Center, Skolkovo 143025, Russia; 5 Department of Physics, Moscow State University, 119991 Moscow, Russia

## Abstract

When a mobile impurity interacts with a many-body system, such as a phonon bath, a polaron is formed. Despite the importance of the polaron problem for a wide range of physical systems, a unified theoretical description valid for arbitrary coupling strengths is still lacking. Here we develop a renormalization group approach for analyzing a paradigmatic model of polarons, the so-called Fröhlich model, and apply it to a problem of impurity atoms immersed in a Bose-Einstein condensate of ultra cold atoms. Polaron energies obtained by our method are in excellent agreement with recent diagrammatic Monte Carlo calculations for a wide range of interaction strengths. They are found to be logarithmically divergent with the ultra-violet cut-off, but physically meaningful regularized polaron energies are also presented. Moreover, we calculate the effective mass of polarons and find a smooth crossover from weak to strong coupling regimes. Possible experimental tests of our results in current experiments with ultra cold atoms are discussed.

A general class of fundamental problems in physics can be described as an impurity particle interacting with a quantum reservoir. This includes Anderson’s orthogonality catastrophe^1^, the Kondo effect^2^, lattice polarons in semiconductors, magnetic polarons in strongly correlated electron systems and the spin-boson model^3^. The most interesting systems in this category can not be understood using a simple perturbative analysis or even self-consistent mean-field (MF) approximations. For example, formation of a Kondo singlet between a spinful impurity and a Fermi sea is a result of multiple scattering processes^4^ and its description requires either a renormalization group (RG) approach^5^ or an exact solution^6^,^7^, or introduction of slave-particles^8^. Another important example is a localization delocalization transition in a spin bath model, arising due to “interactions” between spin flip events mediated by the bath^3^.

While the list of theoretically understood non-perturbative phenomena in quantum impurity problems is impressive, it is essentially limited to one dimensional models and localized impurities. Problems that involve mobile impurities in higher dimensions are mostly considered using quantum Monte Carlo (MC) methods^9^,^10^,^11^. Much less progress has been achieved in the development of efficient approximate schemes. For example a question of orthogonality catastrophe for a mobile impurity interacting with a quantum degenerate gas of fermions remains a subject of active research^12^,^13^.

Recent experimental progress in the field of ultracold atoms brought new interest in the study of impurity problems. Feshbach resonances made it possible to realize both Fermi^14^,^15^,^16^,^17^,^18^,^19^ and Bose polarons^20^,^21^ with tunable interactions between the impurity and host atoms. Detailed information about Fermi polarons was obtained using a rich toolbox available in these experiments. Radio frequency (rf) spectroscopy was used to measure the polaron binding energy and to observe the transition between the polaronic and molecular states^14^. The effective mass of Fermi polarons was studied using measurements of collective oscillations in a parabolic confining potential^15^. Polarons in a Bose-Einstein condensate (BEC) received less experimental attention so far although polaronic effects have been observed in nonequilibrium dynamics of impurities in 1d systems^20^,^21^,^22^.

The goal of this paper is two-fold. Our first goal is to introduce a theoretical technique for analyzing a common class of polaron problems, the so-called Fröhlich polarons. We develop a unified approach that can describe polarons all the way from weak to strong couplings. Our second goal is to apply this method to the problem of impurity atoms immersed in a BEC. We focus on calculating the polaron binding energy and effective mass, both of which can be measured experimentally. For this particular polaron model in a BEC we address the long-standing question how the polaron properties depend on the polaronic coupling strength, and whether a true phase transition exists to a self-trapped regime. Our results suggest a smooth cross-over and do not show any non-analyticity in the accessible parameter range. Moreover we investigate the dependence of the groundstate energy on the ultra-violet (UV) cut-off and point out a logarithmic UV divergence. Considering a wide range of atomic mixtures with tunable interactions^23^ and very different mass ratios available in current experiments^24^,^25^,^26^,^27^,^28^,^29^,^30^,^31^,^32^,^33^,^34^,^35^,^36^,^37^,^38^,^39^,^40^,^41^,^42^,^43^,^44^ we expect that many of our predictions can be tested in the near future. In particular we discuss that currently available technology should make it possible to realize intermediate coupling polarons.

Previously the problem of an impurity atom in a superfluid Bose gas has been studied theoretically using self-consistent T-matrix calculations^45^ and variational methods^46^, and within the Fröhlich model in the weak coupling regime^47^,^48^,^49^, the strong coupling approximation^50^,^51^,^52^,^53^,^54^, the variational Feynman path integral approach^55^,^56^,^57^ and the numerical diagrammatic MC simulations^58^. These four methods predicted sufficiently different polaron binding energies in the regimes of intermediate and strong interactions, see Fig. 1. While the MC result can be considered as the most reliable of them, the physical insight gained from this approach is limited. The method developed in this paper builds upon earlier analytical approaches by considering fluctuations on top of the MF state and including correlations between different modes using the RG approach. We verify the accuracy of this method by demonstrating excellent agreement with the MC results^58^ at zero momentum and for intermediate interaction strengths.

Our method provides new insight into polaron states at intermediate and strong coupling by showing the importance of entanglement between phonon modes at different energies. A related perspective on this entanglement was presented in Ref. 59, which developed a variational approach using correlated Gaussian wavefunctions (CGWs) for Fröhlich polarons. Throughout the paper we will compare our RG results to the results computed with CGWs. In particular, we use our method to calculate the effective mass of polarons, which is a subject of special interest for many physical applications and remains an area of much controversy.

The Fröhlich Hamiltonian represents a generic class of models in which a single quantum mechanical particle interacts with the phonon reservoir of the host system. In particular it can describe the interaction of an impurity atom with the Bogoliubov modes of a BEC^50^,^52^,^56^. In this case it reads (*ħ* = 1)


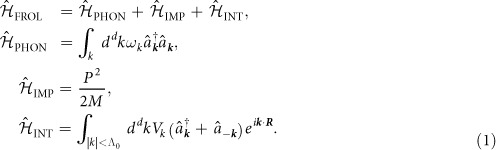


Here *M* denotes the impurity mass and *m* will be the mass of the host bosons, 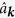 is the annihilation operator of the Bogoliubov phonon excitation in a BEC with momentum ***k***, ***P*** and ***R*** are momentum and position operators of the impurity atom, *d* is the dimensionality of the system and Λ_0_ is a high momentum cutoff needed for regularization. The dispersion of phonon modes of the BEC and their interaction with the impurity atom are given by the standard Bogoliubov expressions^56^


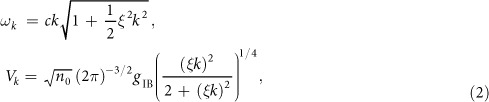


with *n*_0_ being the BEC density and *ξ* = (2*mg*_BB_*n*_0_)^−1/2^ the healing (or coherence) length and *c* = (*g*_BB_*n*_0_/*m*)^1/2^ the speed of sound of the condensate. Here *g*_IB_ denotes the interaction strength between the impurity atom with the bosons, which in the lowest order Born approximation is given by *g*_IB_ = 2*πa*_IB_/*m*_red_, where *a*_IB_ is the scattering length and 
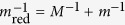
 is the reduced mass of a pair consisting of impurity and bosonic host atoms. Similarly, *g*_BB_ is the boson-boson interaction strength. The analysis of the UV divergent terms in the polaron energy will require us to consider a more accurate cutoff dependent relation between *g*_IB_ and the scattering length *a*_IB_ (see methods).

The Fröhlich Hamiltonian (1) for an impurity atom in a BEC is characterized by only two dimensionless coupling constants when expressing lengths in units of *ξ* and energies in units of *c*/*ξ*. Firstly the mass ration *M*/*m* enters the kinetic energy of the impurity and determines the strength of long-range phonon-phonon interactions mediated by the impurity atom. Secondly, impurity-phonon interactions are determined by the scattering length *a*_IB_ and the BEC density *n*_0_ and can be parametrized by the dimensionless coupling strength^56^


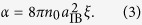


In order to calculate the energy of the impurity atom in the BEC one needs to consider the full expression 
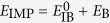
, where 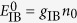 is the MF interaction energy of the impurity with bosons from the condensate, and 
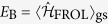
 is the groundstate energy of the Fröhlich Hamiltonian. From now on we will call 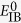 the impurity-condensate interaction energy and *E*_B_ the polaron binding energy. Only *E*_IMP_ is physically meaningful and can be expressed in a universal cutoff independent way using the scattering length *a*_IB_. Precise conditions under which one can use the Fröhlich model to describe the impurity BEC interaction, and parameters of the model for specific cold atoms mixtures are discussed in the discussion section. We point out that the Fröhlich type Hamiltonians (1) are relevant for many systems besides BEC-impurity polarons. Its original and most common use is in the context of electrons coupled to crystal lattice fluctuations in solid state systems^60^. Another important application area is for studying doped quantum magnets, in which electrons and holes are strongly coupled to magnetic fluctuations. Motivated by this generality of the model (1) we will analyze it for a broader range of parameters than may be relevant for the current experiments with ultra cold atoms.

## Results

As a first step we apply the standard Lee-Low-Pines (LLP)^61^ unitary transformation to the Fröhlich Hamiltonian, which separates the total polaron momentum ***P*** as a conserved quantity. Next, we apply a second exact unitary transformation, which displaces the phonons by the MF polaron solution 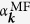
61. This brings the Hamiltonian into the form (see also method section for a self-contained derivation)


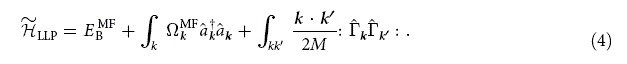


Here 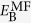 is the polaron binding energy obtained from MF theory and the MF phonon dispersion is denoted by 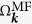. MF polaron theory was formulated for this problem in^48^, and we give a self-contained summary in the methods section. Moreover we defined 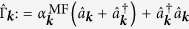, : ... : stands for normal-ordering and we introduced the short-hand notation 
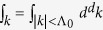
.

### RG Analysis

In this section we provide the RG solution of the Hamiltonian (4), describing quantum fluctuations on top of the MF polaron state. We begin with a dimensional analysis of different terms in (4) in the long wavelength limit, which establishes that only one of the interaction terms is marginal and all others are irrelevant. Then we present the RG flow equations for parameters of the model, including the expression for the polaron binding energy. We note that electron-phonon interactions of the Fröhlich type, see Eq. (1), have been treated before using a different RG formalism^62^,^63^. There, phonons were integrated out exactly, and in contrast to the method introduced below all information about phonon correlations in the polaron cloud was lost.

Our approach to the RG treatment of the model (4) is similar to the “poor man’s RG” in the context of the Kondo problem. We use Schrieffer-Wolff type transformation to integrate out high energy phonons in a thin shell in momentum space near the cutoff, Λ − *δ*Λ < *k* < Λ, using 1/Ω_***k***_ as a small parameter (Ω_***k***_ being the frequency of phonons in the thin momentum-shell, where initially 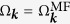). This transformation renormalizes the effective Hamiltonian for the low energy phonons. Iterating this procedure we get a flow of the effective Hamiltonian with the cutoff parameter Λ.

To analyze whether the system flows to strong or weak coupling in the long wavelength limit |***k***|*ξ* ≪ 1 we consider scaling dimensions of different operators in (4). We fix the dimension of 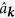 using the condition that 
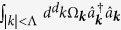
 is scale invariant. In the long wavelength limit phonons have linear dispersion Ω_***k***_ ∝ |***k***|, which requires scaling of 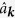 as 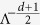. Scaling dimensions of different contributions to the interaction part of the Hamiltonian (26) is shown in Table 1. We observe that, as the cutoff scale tends to zero, most terms are irrelevant and only the quartic term 
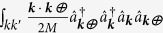
 is marginal. As we demonstrate below, this term is marginally irrelevant, i.e. in the process of RG flow the impurity mass *M* flows to large values. This feature provides justification for doing the RG perturbation expansion with 1/*M* as the interaction parameter. Also, an irrelevance of the interaction under the RG flow physically means that at least slow phonons in the system are Gaussian. This provides an insight why the variational correlated Gaussian wavefunctions^59^ are applicable for the Fröhlich Hamiltonian under consideration.

By starting from the Hamiltonian (4) and calculating its RG flow we find that the general RG Hamiltonian can be written in the following way,


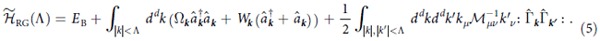


Note that the interaction is now characterized by a general tensor 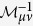 [the indices *μ* = *x*, *y*, *z*, ... label cartesian coordinates and they are summed over when occurring twice], where the anisotropy originates from the conserved total momentum of the polaron ***P*** = *P**e***_*x*_, breaking the rotational symmetry of the system. Due to the cylindrical symmetry of the problem, the mass tensor has the form 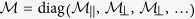, and we find different flows for the longitudinal and the transverse components of the mass tensor. While 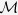 can be interpreted as the (tensor-valued) renormalized mass of the impurity, it should not be confused with the mass of the polaron. The first line of Eq. (5) describes the diagonal quadratic part of the renormalized phonon Hamiltonian. It is also renormalized compared to the MF expression 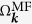,


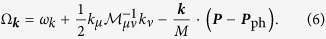


The momentum carried by the phonon-cloud, ***P***_ph_, acquires an RG flow, describing corrections to the MF result 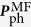. In addition there is a term linear in the phonon operators, weighted by


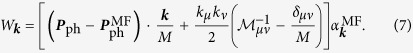


By comparing Eq. (5) to Eq. (4) we obtain the initial conditions for the RG, starting at the original UV cutoff Λ_0_ where 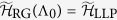,


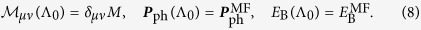


We derive the following flow equations for the parameters in 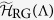 (see methods for details),


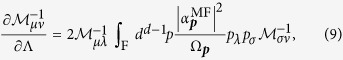



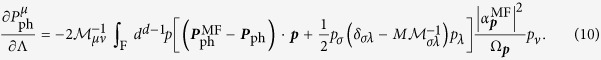


Here we use the notation 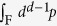 for the integral over the *d* − 1 dimensional surface defined by momenta of length |***p***| = Λ. The energy correction to the binding energy of the polaron beyond MF theory, 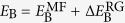, is given by


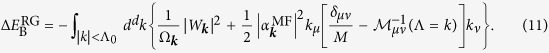


Note that, in this expression, we evaluated the renormalized impurity mass 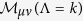 at a value of the running cutoff Λ = *k* given by the integration variable *k* = |***k***|. Similarly, it is implicitly assumed that ***P***_ph_(***k***) and 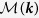 appearing in the expressions for *W*_***k***_ and Ω_***k***_, see Eqs (7) and (6), are evaluated at Λ = *k*.

**Figure 1:**
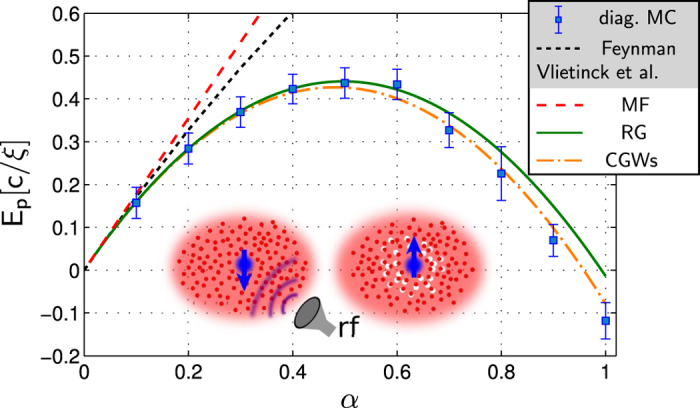
By applying a rf-pulse to flip a non-interacting (left inset) into an interacting impurity state (right inset) a Bose polaron can be created in a BEC. From the corresponding rf-spectrum the polaron groundstate energy can be obtained. In the main plot we compare polaronic contributions to the energy *E*_p_ (as defined in Eq. (25)) predicted by different models, as a function of the coupling strength *α*. Our results (RG) are compared to calculations with correlated Gaussian wavefunctions (CGWs)^59^, MC calculations by Vlietinck *et al.*^58^, Feynman variational calculations by Tempere *et al.*^56^ and MF theory. We used the standard regularization scheme to cancel the leading power-law divergence of *E*_p_. However, to enable comparison with the MC data, we did *not* regularize the UV log-divergence reported in this paper. Hence the result is sensitive to the UV cutoff chosen for the numerics, and we used the same value Λ_0_ = 2000/*ξ* as in^58^. Other parameters are *M*/*m* = 0.263158 and *P* = 0.

**Figure 2:**
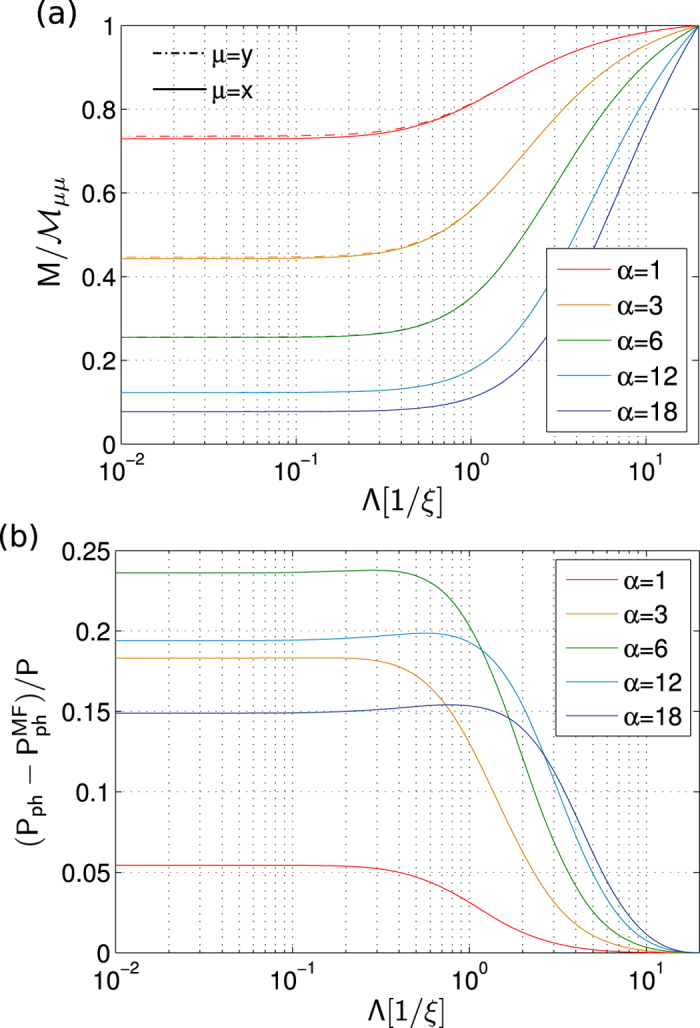
Typical RG flows of the (inverse) renormalized impurity mass 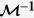 (**a**) and the excess phonon momentum 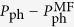 along the direction of the system momentum *P* (**b**). Results are shown for different coupling strengths *α* (defined in Eq. (3)) and we used parameters *M*/*m* = 0.3, *P*/*Mc* = 0.5 and Λ_0_ = 20/*ξ* in *d* = 3 dimensions.

Figure 2 shows typical RG flows of 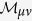 and 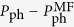. For 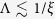 we observe quick convergence of these coupling constants. One can see comparison of the MC and RG calculations^58^ for the polaron binding energy at momentum *P* = 0 in Fig. 1. The agreement is excellent for a broad range of interaction strengths. We will further discuss these results below.

### Cutoff dependence

In a three dimensional system (*d* = 3), examination of the binding energy of the polaron *E*_B_ shows that it is UV divergent. To leading order it scales linearly with the UV cut-off, *E*_B_ ~ Λ_0_. This divergence is known from MF polaron theory, and it can be regularized by using the second order Lippman-Schwinger equation to relate the scattering length *a*_IB_ to the interaction strength *g*_IB_ which determines the impurity-condensate interaction (see methods for a brief review). From our RG protocol, in addition, we obtain a sub-leading logarithmic UV divergence, *E*_B_ ~ −log(Λ_0_*ξ*). To obtain cut-off independent polaron energies *E*_IMP_ we developed a regularization scheme which cancels the log-divergence, by including beyond-MF effects in the Lippman-Schwinger equation. The details of this scheme will be presented in a separate publication^64^. All other observables discussed in this paper, such as the effective polaron mass, are UV convergent, and thus no regularization will be required.

### Polaron Energy

**Figure 3:**
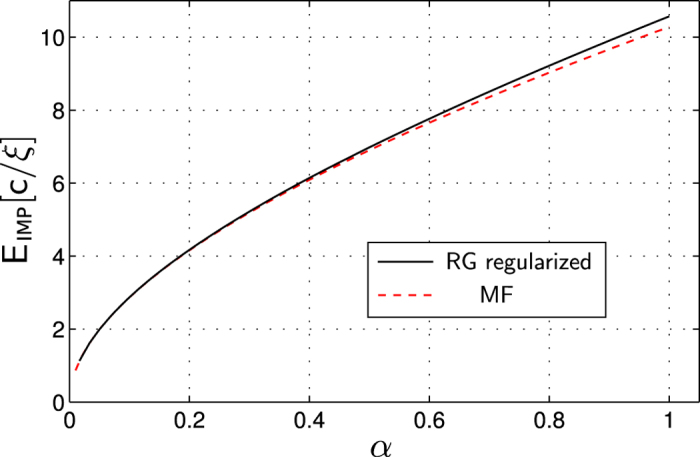
The impurity energy *E*_IMP_(*α*), which can be measured in a cold atom setup using rf-spectroscopy, is shown as a function of the coupling strength *α*. Our prediction from the RG is given by the solid black line, representing the fully regularized impurity energy. We compare our results to MF theory (dashed). Note that, although MF yields a strict upper variational bound on the binding energy *E*_B_, the MF impurity energy *E*_IMP_ is below the RG prediction because the impurity-condensate interaction 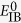 was treated more accurately in the latter case. We used parameters *M*/*m* = 0.26316, Λ_0_ = 2000/*ξ*, *P* = 0 and set the BEC density to *n*_0_ = *ξ*^−3^.

The impurity energy (calculated from the RG and using our regularization scheme) is shown in Fig. 3 as a function of the polaronic coupling strength *α*. The resulting energy is close to, but slightly above, the MF energy. [This result might be surprising at first glance, because MF polaron theory relies on a variational principle and thus yields an upper bound for the groundstate energy. However, this bound holds only for the binding energy *E*_B_, defined as the groundstate energy of the Fröhlich Hamiltonian, and not for the entire impurity Hamiltonian including the condensate-impurity interaction.] We thus conclude that the large deviations observed in Fig. 1 of beyond MF theories (MC, RG, variational) from MF are merely an artifact of the logarithmic UV divergence which was not properly regularized. We note that this also explains — at least partly — the unexpectedly large deviations of Feynman’s variational approach from the numerically exact MC results, reported in^58^, since Feynman’s model does not capture the log-divergence^58^.

Our results have important implications for experiments. The relatively small difference in energy between MF and (properly regularized) RG in Fig. 3 demonstrates that a measurement of the impurity energy alone does not allow to discriminate between uncorrelated MF theories and extensions thereof (like RG or MC). Yet such a measurement would still be significant as a consistency check of our regularization scheme. To find smoking gun signatures for beyond MF behavior, i.e. for a regime dominated by quantum fluctuations, other observables are required such as the effective polaron mass, which we discuss next.

### Effective Polaron Mass

**Figure 4:**
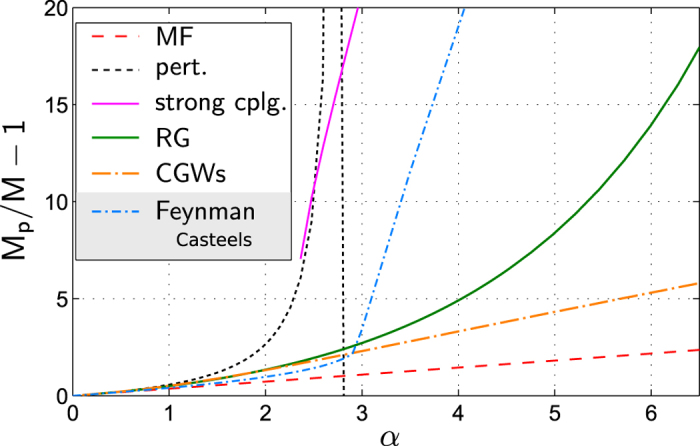
The polaron mass *M*_p_ (in units of *M*) is shown as a function of the coupling strength *α*. We compare our results (RG) to variational calculations using CGWs^59^ and MF calculations, strong coupling theory^54^ and Feynman’s variational path-integral approach^55^. The path-integral results were obtained by Wim Casteels^57^, and we are grateful to him for providing this data to us. We used parameters *M*/*m* = 0.26, Λ_0_ = 200/*ξ* and set *P*/*Mc* = 0.01.

In Fig. 4 we show the polaron mass calculated using several different approaches. In the weak coupling limit *α* → 0 the polaron mass can be calculated perturbatively in *α*, and the lowest-order result is shown in Fig. 4. We observe that in this limit, all approaches follow the same line which asymptotically approaches the perturbative result (as *α* → 0). The only exception is the strong coupling Landau-Pekkar approach, which yields a self-trapped polaron solution only beyond a critical value of *α*^54^.

For larger values of *α*, MF theory sets a lower bound for the polaron mass. Naively this is expected, because MF theory does not account for quantum fluctuations due to couplings between phonons of different momenta. These fluctuations require additional correlations to be present in beyond MF wavefunctions, like e.g. in our RG approach, which should lead to an increased polaron mass. Indeed, for intermediate couplings 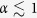 the RG, as well as the variational approach using CGWs, predict a polaron mass 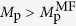 which is considerably different from the MF result^48^.

In Fig. 4 we present the most interesting aspect of our analysis, which is related to the nature of the cross-over from weak to strong coupling polaron regime. While Feynman’s variational approach predicts a sharp transition, the RG and CGWs results show no sign of any discontinuity in the accessible parameter range. Instead they suggest a smooth cross-over from one into the other regime. This is also expected on general grounds, and rigorous proofs were given for generic polaron models in Refs 65, 66. The proofs do not apply to the Fröhlich Hamiltonian in a BEC, see Eq. (1), however. Interestingly, for closely related Fröhlich polarons with acoustic phonons, indications for a true phase transition were found in the solid-state context^67^. It is possible that the sharp crossover obtained using Feynman’s variational approach is an artifact of the limited number of parameters used in the variational action. It would be interesting to consider a more general class of variational actions^20^,^68^.

**Figure 5:**
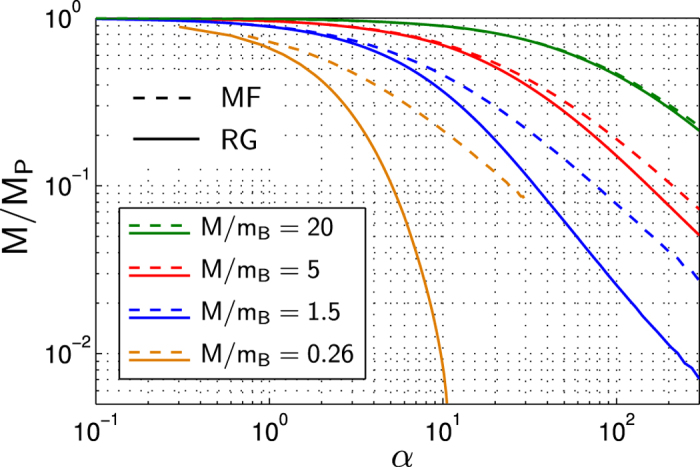
The inverse polaron mass *M*/*M*_p_ is shown as a function of the coupling strength *α*, for various mass ratios *M*/*m*. We compare MF (dashed) to RG (solid) results. The parameters are Λ_0_ = 2000/*ξ* and we set *P*/*Mc* = 0.01 in the calculations.

**Figure 6:**
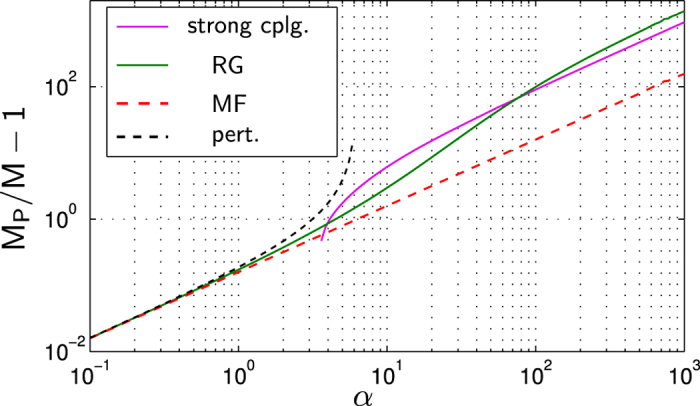
The polaron mass *M*_p_/*M* is shown as a function of the coupling strength for an impurity of mass *M* = *m* equal to the boson mass. We compare the asymptotic perturbation and strong coupling theories with MF and RG, which can be formulated for all values of the coupling strength. We used parameters Λ_0_ = 200/*ξ* and *P*/*Mc* = 0.01.

In Fig. 4 we calculated the polaron mass in the strongly coupled regime, where *α* ≫ 1 and the impurity-boson mass ratio *M*/*m* = 0.26 is small. It is also instructive to see how the system approaches the integrable limit *M* → ∞ when it becomes exactly solvable^48^. Figure 5 shows the (inverse) polaron mass as a function of *α* for different mass ratios *M*/*m*. For *M* ≫ *m*, as expected, the corrections from the RG are negligible and MF theory is accurate. When the mass ratio *M*/*m* approaches unity, we observe deviations from the MF behavior for couplings above a critical value of *α* which depends on the mass ratio. Remarkably, for very large values of *α* the mass predicted by the RG follows the same power-law as the MF solution, with a different prefactor. This can be seen more clearly in Fig. 6, where the case *M*/*m* = 1 is presented. This behavior can be explained from strong coupling theory. As shown in^54^ the polaron mass in this regime is proportional to *α*, as is the case for the MF solution. However prefactors entering the expressions for the weak coupling MF and the strong coupling masses are different.

To make this more precise, we compare the MF, RG and strong coupling polaron masses for *M*/*m* = 1 in Fig. 6. We observe that the RG smoothly interpolates between the weak coupling MF and the strong coupling regime. While the MF solution is asymptotically recovered for small *α* → 0 (by construction), this is not strictly true on the strong coupling side. Nevertheless, the observed value of the RG polaron mass in Fig. 6 at large *α* is closer to the strong coupling result than to the MF theory.

Now we return to the discussion of the polaron mass for systems with a small mass ratio *M*/*m* < 1. In this case Fig. 5 suggests that there exists a large regime of intermediate coupling, where neither strong coupling nor MF theory can describe the qualitative behavior of the polaron mass. This is demonstrated in Fig. 4, where our RG approach predicts values for the polaron mass midway between MF and strong coupling, for a wide range of couplings. In this intermediate-coupling regime, the impurity is constantly scattered on phonons, leading to strong correlations between them.

Thus measurements of the polaron mass rather than the binding energy should be a good way to discriminate between different theories describing the Fröhlich polaron at intermediate couplings. Quantum fluctuations manifest themselves in a large increase of the effective mass of polarons, in strong contrast to the predictions of the MF approach based on the wavefunction with uncorrelated phonons. Experimentally, both the quantitative value of the polaron mass, as well as its qualitative dependence on the coupling strength can provide tests of our theory. The mass of the Fermi polaron has successfully been measured using collective oscillations of the atomic cloud^15^, and we are optimistic that similar experiments can be carried out with Bose polarons in the near future. Alternatively, momentum resolved radio-frequency spectroscopy can be used to measure the mass of the polaron, see e.g.^48^. If imbalanced atomic mixtures are used, the polaron-polaron interactions need to be sufficiently weak to prevent the system from phase-separation, as discussed in Ref. 69using the strong-coupling approximation.

## Discussion

Now we discuss conditions under which the Fröhlich Hamiltonian can be used to describe impurities in ultra cold quantum gases. We also present typical experimental parameters and show that the intermediate coupling regime *α* ~ 1 can be reached with current technology. Possible experiments in which the effects predicted in this paper could be observed are also discussed.

To derive the Fröhlich Hamiltonian Eq. (1) for an impurity atom immersed in a BEC^52^,^56^, the Bose gas is described in Bogoliubov approximation, valid for weakly interacting BECs. Then the impurity interacts with the elementary excitations of the condensate, which are Bogoliubov phonons. In writing the Fröhlich Hamiltonian to describe these interactions, we included only terms that are linear in the Bogoliubov operators. This implicitly assumes that the condensate depletion Δ*n* caused by the impurity is much smaller than the original BEC density, Δ*n*/*n*_0_ = 1, giving rise to the condition^52^


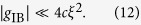


When this condition is not fulfilled, other interesting phenomena like the formation of a bubble polaron^70^ can be expected which go beyond the physics described by the Fröhlich model.

To reach the intermediate coupling regime of the Fröhlich model, coupling constants *α* larger than one 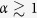 are required (for mass ratios *M*/*m* ≃ 1 of the order of one). This can be achieved by a sufficiently large impurity-boson interaction strength *g*_IB_, which however means that condition (12) becomes more stringent. Now we discuss under which conditions both 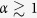 and Eq. (12) can simultaneously be fulfilled. To this end we express both equations in terms of experimentally relevant parameters *a*_BB_ (boson-boson scattering length), *m* and *M* which are assumed to be fixed, and we treat the BEC density *n*_0_ and the impurity-boson scattering length *a*_IB_ as experimentally tunable parameters. Using the first-order Born approximation result *g*_IB_ = 2*πa*_IB_/*m*_red_ Eq. (12) reads


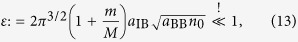


and similarly the polaronic coupling constant can be expressed as


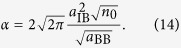


Both *α* and *ε* are proportional to the BEC density *n*_0_, but while *α* scales with 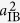, *ε* is only proportional to *a*_IB_. Thus to approach the strong coupling regime *a*_IB_ has to be chosen sufficiently large, while the BEC density has to be small enough in order to satisfy Eq. (13). When setting *ε* = 0.3 ≪ 1 and assuming a fixed impurity-boson scattering length *a*_IB_, we find an upper bound for the BEC density,


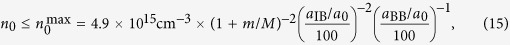


where *a*_0_ denotes the Bohr radius. For the same fixed value of *a*_IB_ the coupling constant *α* takes a maximal value


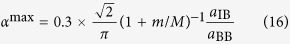


compatible with condition (12).

Before discussing how Feshbach resonances allow to reach the intermediate coupling regime, we estimate values for *α*^max^ and 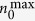 for typical background scattering lengths *a*_IB_. Despite the fact that these *a*_IB_ are still rather small, we find that keeping track of condition (13) is important. To this end we consider two experimentally relevant mixtures, (i) ^87^Rb (majority) -^41^K^20^,^29^ and (ii) ^87^Rb (majority) -^133^Cs^25^,^28^. For both cases the boson-boson scattering length is *a*_BB_ = 100*a*_0_^23^,^24^ and typical BEC peak densities realized experimentally are *n*_0_ = 1.4 × 10^14^cm^−3^ 29. In the first case (i) the background impurity-boson scattering length is *a*_Rb−K_ = 284*a*_0_^23^, yielding *α*_Rb−K_ = 0.18 and *ε* = 0.21 ≪ 1. By setting *ε* = 0.3 for the same *a*_Rb−K_, Eq. (15) yields an upper bound for the BEC density 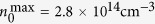 above the value of *n*_0_, and a maximum coupling constant 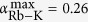. For the second mixture (ii) the background impurity-boson scattering length *a*_Rb−Cs_ = 650*a*_0_^28^ leads to *α*_Rb−Cs_ = 0.96 but *ε* = 0.83 < 1. Setting *ε* = 0.3 for the same value of *a*_Rb−Cs_ yields 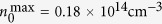 and 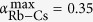. We thus note that already for small values of 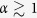, Eq. (13) is *not* automatically fulfilled and has to be kept in mind.

The impurity-boson interactions, i.e. *a*_IB_, can be tuned by the use of an inter-species Feshbach resonance^23^, available in a number of experimentally relevant mixtures^26^,^31^,^37^,^38^,^39^,^42^,^43^. In this way, an increase of the impurity-boson scattering length by more than one order of magnitude is realistic. In Table 2 we show the maximally achievable coupling constants *α*^max^ for several impurity-boson scattering lengths and imposing the condition *ε* < 0.3. We consider the two mixtures from above (^87^Rb − ^41^K and ^87^Rb − ^133^Cs), where broad Feshbach resonances are available^20^,^26^,^37^,^38^. We find that coupling constants *α* ~ 1 in the intermediate coupling regime can be realized, which are compatible with the Fröhlich model and respect condition (12). The required BEC densities are of the order *n*_0_ ~ 10^13^ cm^−3^, which should be achievable with current technology. Note that when Eq. (12) would not be taken into account, couplings as large as *α* ~ 100 would be possible, but then *ε* ~ 8 ≫ 1 indicates the importance of the phonon-phonon scatterings neglected in the Fröhlich model. Bose polarons in such close vicinity to a Feshbach resonance have also been discussed in Refs 45,46.

## Methods

### Fröhlich Hamiltonian in the impurity frame

The Hamiltonian (1) describes a translationally invariant system. It is convenient to perform the LLP transformation^61^ that separates the system into decoupled sectors of conserved total momentum,


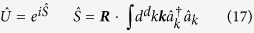



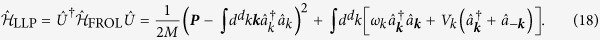


The transformed Hamiltonian (18) does no longer contain the impurity position operator ***R***. Thus ***P*** in equation (18) is a conserved net momentum of the system and can be treated as a 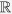-number (rather than a hermitian operator). Alternatively, the transformation (17) is commonly described as going into the impurity frame, since the term describing boson scattering on the impurity in (18) is obtained from the corresponding term in (1) by setting ***R*** = 0. The Hamiltonian (18) has only phonon degrees of freedom but they now interact with each other. This can be understood physically as a phonon-phonon interaction, mediated by an exchange of momentum with the impurity atom. This impurity-induced interaction between phonons in Eq. (1) is proportional to 1/*M*. Thus in our analysis of the polaron properties, which is based on the LLP transformed Fröhlich Hamiltonian, we will consider 1/*M* as controlling the interaction strength.

### Review of the mean field approximation

In this section we briefly review the MF approach to the polaron problem, which provides an accurate description of the system when quantum fluctuations do not play an important role, e.g. for weak coupling *α*  1 or large impurity mass. We discuss how one should regularize the MF interaction energy, which is UV divergent for *d* ≥ 2. To set the stage for subsequent beyond MF analysis of the polaron problem, we derive the Hamiltonian that describes fluctuations around the MF state.

The MF approach to calculating the ground state properties of (18) is to consider a variational wavefunction in which all phonons are taken to be in a coherent state^61^. The MF variational wavefunction reads


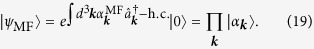


It becomes exact in the limit of an infinitely heavy (i.e. localized) impurity. Energy minimization with respect to the variational parameters *α*_***k***_ gives


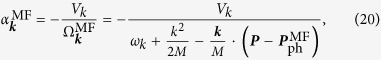


where 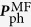 is the momentum of the system carried by the phonons. It has to be determined self-consistently from the solution (20),
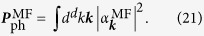
The MF character of the wave function (19) is apparent from the fact that it is a product of wave functions for individual phonon modes. Hence it contains neither entanglement nor correlations between different modes. The only interaction between modes is through the selfconsistency equation (21).

Properties of the MF solution have been discussed extensively in Refs 48,61,71. Here we reiterate only one important issue related to the high energy regularization of the MF energy^45^,^48^,^56^. In *d* ≥ 2 dimensions the expression for the MF energy,


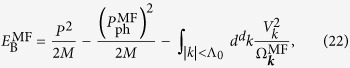


is UV divergent as the high momentum cutoff Λ_0_ is sent to infinity. In order to regularize this expression we recall that the physical energy of the impurity is a sum of 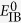 and the polaron binding energy *E*_B_. If we use the leading order Born approximation to express *g*_IB_ = 2*πa*_IB_/*m*_red_, we observe that the MF polaron energy has contributions starting with the second order in *a*_IB_. Consistency requires that the impurity-condensate interaction energy 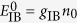 is computed to order 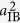. The Lippman-Schwinger equation provides the relation between the microscopic interaction *g*_IB_, the cutoff Λ_0_ and the physical scattering length *a*_IB_^72^


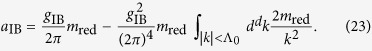


To second order in *a*_IB_ one has


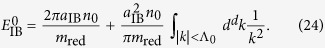


Now we recognize that in the physically meaningful impurity energy 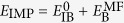 the UV divergence cancels between 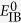 and 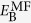.

Thus separation of the impurity energy into the impurity-condensate interaction 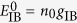 and the binding energy *E*_B_ is not physically meaningful. A decomposition of the impurity energy in orders of the scattering length *a*_IB_, on the other hand, is well defined,


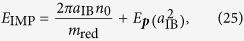


where the second-order term is referred to as the polaronic energy of the impurity^56^.

The main shortcoming of the MF ansatz (19) is that it discards correlations between phonons with different energies and at different momenta. In this paper we develop a method that allows us to go beyond the MF solution (19) and include correlations between modes. In the following we demonstrate how this can be accomplished, and discuss physical consequences of phonon correlations. To simplify the subsequent discussion we perform a unitary transformation that shifts the phonon variables in Eq. (18) by the amount corresponding to the MF solution,


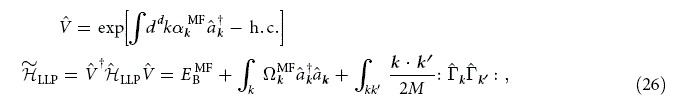


see Eq. (4) in the main text. Note that the absence of terms linear in 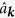 in the last equation reflects the fact that 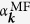 correspond to the MF (saddle point) solution. We emphasize that (26) is an exact representation of the original Fröhlich Hamiltonian, where the operators 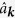 describe quantum fluctuations around the MF polaron.

### Derivation of RG flow equations

We now derive the RG flow equations of the general Hamiltonian (5), which should be supplemented by the initial conditions in Eq. (8). To this end we separate phonons into “fast” ones with momenta ***p*** and “slow” ones with momenta ***k***, according to Λ − *δ*Λ < |***p***| < Λ and |***k***| ≤ Λ − *δ*Λ. Then the Hamiltonian (5) can be split into


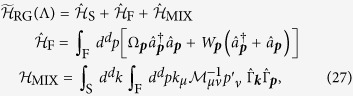


where we use the short-hand notations 
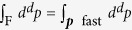
 and 
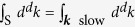
. The slow-phonon Hamiltonian 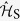 is given by Eq. (5) except that all integrals only go over slow phonons, 
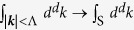
. In 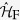 we do not have a contribution due to the interaction term since it would be proportional to *δ*Λ^2^ and we will consider the limit *δ*Λ → 0. We can obtain intuition into the nature of the transformation needed to decouple fast from slow phonons, by observing that for the fast phonons the Hamiltonian (27) is similar to a harmonic oscillator in the presence of an external force (recall that 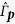 contains only linear and quadratic terms in 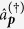). This external force is determined by the state of slow phonons. Thus it is natural to look for the transformation as a shift operator for the fast phonons,


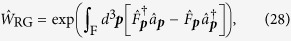


with coefficients 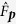 depending on the slow phonons only, i.e. 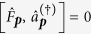. One can check that taking


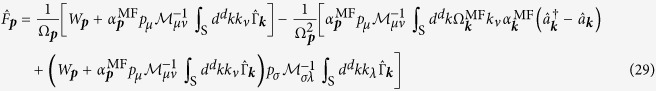


eliminates non-diagonal terms in 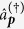 up to second order in 1/Ω_***p***_. After the transformation we find


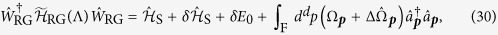



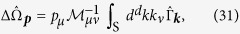



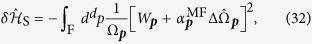



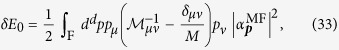


which is valid up to corrections of order 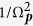 or *δ*Λ^2^. The last equation describes a change of the zero-point energy *δE*_0_ of the impurity in the potential created by the phonons, and it is caused by the RG flow of the impurity mass. To obtain this term we have to carefully treat the normal-ordered term 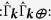 in Eq. (5). [The following relation is helpful to perform normal-ordering, 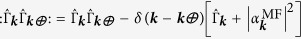.] We will show later that this contribution to the polaron binding energy is crucial because it leads to a UV divergence in *d* ≥ 3 dimensions.

From the last term in Eq. (30) we observe that the ground state |gs〉 of the Hamiltonian is obtained by setting the occupation number of high energy phonons to zero, 
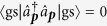
. Then from Eq. (32) we read off the change in the Hamiltonian for the low energy phonons. From the form of the operator 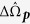 in Eq. (31) one easily shows that the new Hamiltonian 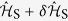 is of the universal form 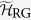, but with renormalized couplings. This gives rise to the RG flow equations for the parameters in 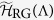 presented in Eqs (9, 10, 11).

### Calculation of the polaron mass

In this section we provide a few specifics on how we calculate the polaron mass. We relate the average impurity velocity to the polaron mass *M*_p_ and obtain


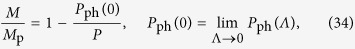


where *M* is the bare impurity mass. The argument goes as follows. The average polaron velocity is given by *v*_p_ = *P*/*M*_p_. The average impurity velocity *v*_I_, which by definition coincides with the average polaron velocity *v*_I_ = *v*_p_, can be related to the average impurity momentum *P*_I_ by *v*_I_ = *P*_I_/*M*. Because the total momentum is conserved, *P* = *P*_ph_ + *P*_I_, we thus have *P*/*M*_p_ = *v*_p_ = *v*_I_ = (*P* − *P*_ph_)/*M*. Because the total phonon momentum *P*_ph_ in the polaron groundstate is obtained from the RG by solving the RG flow equation in the limit Λ → 0, we have *P*_ph_ = *P*_ph_(0) as defined above, and Eq. (34) follows. We note that in the MF case this result is exact and can be proven rigorously, see^48^. This is also true for the variational approach based on CGWs^59^.

## Additional Information

**How to cite this article**: Grusdt, F. *et al.* Renormalization group approach to the FrÖhlich polaron model: application to impurity-BEC problem. *Sci. Rep.* **5**, 12124; doi: 10.1038/srep12124 (2015).

## Figures and Tables

**Table 1:** Dimensional analysis is performed by power-counting of the different terms describing quantum fluctuations around the MF polaron state.

operator	scaling (Λξ ≫ 1)
*â*_***k***_	Λ^−(*d*+1)/2^
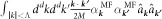	Λ^*d*^
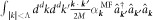	Λ^*d*/2^
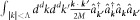	Λ^0^ = 1

We fixed the scaling dimension of *â*_***k***_ such that 
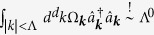
 is scale-invariant.

**Table 2:** Experimentally the impurity-boson scattering length *a*_IB_ can be tuned by more than one order of magnitude using a Feshbach-resonance.

*a*_Rb−K_/*a*_0_	284.	994.	1704.	2414.	3124.	3834.
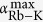	0.26	0.91	1.6	2.2	2.9	3.5
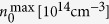	2.8	0.23	0.078	0.039	0.023	0.015
*a*_Rb−Cs_/*a*_0_	650.	1950.	3250.	4550.	5850.	7150.
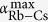	0.35	1.0	1.7	2.4	3.1	3.8
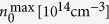	0.18	0.02	0.0073	0.0037	0.0022	0.0015

We consider two mixtures (^87^Rb − ^41^K, top and ^87^Rb − ^133^Cs, bottom) and show the maximally allowed BEC density 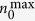 along with the largest achievable coupling constant *α*^max^ compatible with the Fröhlich model, using different values of *a*_IB_.
